# Clinical characteristics of pulmonary nocardiosis in patients with and without bronchiectasis: a single-center retrospective comparative study

**DOI:** 10.3389/fcimb.2026.1910396

**Published:** 2026-09-16

**Authors:** Songsong Yu, Zhenru Ma, Kun Yang, Qianyu Zhang, Qiuhong Fang

**Affiliations:** 1 Department of Emergency, Emergency and Critical Care Medical Center, Beijing Shijitan Hospital, Capital Medical University, Beijing, China; 2 Department II of Internal Medicine, Beijing Changping Nankou Hospital, Beijing, China; 3 Department of Respiratory and Critical Care Medicine, Beijing Chaoyang Hospital, Capital Medical University, Beijing, China

**Keywords:** *Nocardia*, pulmonary nocardiosis, bronchiectasis, respiratory failure, clinical characteristics

## Abstract

**Background:**

Bronchiectasis is a common structural lung disease associated with pulmonary nocardiosis (PN), but the differences between PN patients with and without bronchiectasis remain insufficiently characterized.

**Methods:**

We conducted a single-center retrospective study of hospitalized patients with PN at Beijing Chaoyang Hospital from January 2012 to December 2024. Patients were grouped by bronchiectasis status, and their clinical, radiological, and microbiological features were compared. Multivariable logistic regression was used to identify factors independently associated with bronchiectasis.

**Results:**

Among 154 patients, 86 (55.8%) had bronchiectasis. Of 162 *Nocardia* isolates, *N. cyriacigeorgica*, *N. farcinica*, and *N. abscessus* were the most commonly identified species. Patients with bronchiectasis more frequently had hemoptysis (40.7% vs. 13.2%, *P*<0.001) and tree-in-bud signs (11.6% vs. 2.9%, *P*=0.046), but less frequently had respiratory failure (10.5% vs. 26.5%, *P*=0.022) and had shorter hospital stays (12.62 ± 9.01 vs. 16.96 ± 10.84 days, *P*=0.007). Patients without bronchiectasis more frequently had type 2 diabetes, bronchial asthma, and several parenchymal or pleural imaging findings. In multivariable analysis, hemoptysis was positively associated with bronchiectasis [adjusted odds ratio (OR), 5.407; 95% confidence interval (CI), 2.185–13.382], whereas type 2 diabetes (adjusted OR, 0.320; 95% CI, 0.115–0.888) and bronchial asthma (adjusted OR, 0.166; 95% CI, 0.030–0.928) were negatively associated with bronchiectasis.

**Conclusion:**

PN showed distinct clinical and radiological patterns according to bronchiectasis status. Greater diagnostic attention may be warranted in patients without bronchiectasis.

## 1 Introduction

*Nocardia* species are aerobic, weakly acid-fast, Gram-positive filamentous bacteria that are widely distributed in soil, water, dust, and decaying organic matter (Brown-Elliott et al., 2006; Traxler et al., 2022). Human infection usually occurs through inhalation or direct inoculation, and pulmonary nocardiosis (PN) is the most common clinical form (Ott et al., 2019; Duggal and Chugh, 2020; Mehrabadi et al., 2020). Nocardiosis is a rare, opportunistic disease with an estimated incidence generally below 1 case per 100,000 population per year (Duggal and Chugh, 2020), although its reported incidence has increased in some regions (Tremblay et al., 2011). A recent global systematic analysis of 9,750 reported cases showed that the number of reported nocardiosis cases has increased markedly since 2000 and that the overall all-cause mortality reached 19.8%, highlighting the growing clinical burden of this disease (Du et al., 2025). The disease mainly affects immunocompromised hosts and patients with relevant underlying conditions, including patients with malignancy, diabetes, organ transplantation, long-term corticosteroid use, or other immunosuppressive conditions (Duggal and Chugh, 2020; Lynch et al., 2020; Traxler et al., 2022). A retrospective case-control study showed that immunosuppressive pharmacotherapy, particularly systemic corticosteroid therapy, was strongly associated with nocardiosis, especially PN and nocardiosis in patients with chronic pulmonary disease (Margalit et al., 2020). In immunocompetent individuals, PN is more commonly associated with preexisting structural lung diseases, such as chronic obstructive pulmonary disease, bronchiectasis, or previous pulmonary tuberculosis (Fujita et al., 2016; Traxler et al., 2022). Notably, PN remains clinically challenging because its symptoms and radiological findings are nonspecific and may mimic other pulmonary diseases (Martínez et al., 2008; Rouzaud et al., 2018). Moreover, *Nocardia* may require prolonged culture incubation and specialized microbiological or molecular methods for species-level identification, which can delay diagnosis and contribute to clinical deterioration (Martínez et al., 2008; Lebeaux et al., 2014; Fujita et al., 2016; Ninan et al., 2022). Therefore, better characterization of its clinical features, risk profiles, microbiological distribution, and outcomes is essential for improving early diagnosis and timely treatment.

Bronchiectasis has been identified as the most common underlying disease among PN patients, with some cohorts reporting a prevalence of more than 30%, and may serve as an important risk factor for *Nocardia* colonization (Woodworth et al., 2017; Zhong et al., 2022; Chen et al., 2024). This association may be partly explained by the impaired mucociliary clearance and chronic airway inflammation in bronchiectasis, which provide a susceptible airway environment for *Nocardia* colonization or infection (Yetmar et al., 2025). However, the relationship between bronchiectasis and PN is complex. On the one hand, the chronic symptoms of bronchiectasis, such as cough, expectoration, hemoptysis, and recurrent exacerbations, may overlap with those of PN and obscure early recognition (Singh et al., 2016). Conversely, persistent or insufficiently treated *Nocardia* infection may amplify the vicious cycle of airway infection, impaired mucociliary clearance, and structural lung damage, thereby potentially worsening bronchiectasis-related disease progression (Venditto et al., 2025). Notably, a recent study showed that patients with and without bronchiectasis differed in clinical characteristics, species distribution, and antimicrobial susceptibility profiles, suggesting that this subgroup should be analyzed separately (Han et al., 2024). Therefore, comparing PN patients with and without bronchiectasis is clinically important for recognizing subgroup-specific disease patterns and promoting early and accurate diagnosis.

In this study, we retrospectively analyzed hospitalized PN patients at a tertiary hospital over a 13-year period and compared the clinical characteristics, microbiological profiles, and outcomes between patients with and without bronchiectasis. We aimed to characterize the subgroup-specific features of PN and to provide clinical evidence for earlier recognition of this disease.

## 2 Methods

### 2.1 Study design and participants

This was a single-center retrospective comparative study. Patients hospitalized with PN-related diagnoses in the Department of Respiratory and Critical Care Medicine, Beijing Chaoyang Hospital, Capital Medical University, between January 1, 2012 and December 31, 2024 were screened. The inclusion criteria were as follows: 1) hospitalization during the study period; 2) diagnosis of PN; and 3) availability of complete clinical data for analysis. PN was diagnosed based on compatible clinical manifestations, such as fever, cough, expectoration, hemoptysis, chest pain, or dyspnea; chest imaging showing pulmonary infectious lesions; and microbiological isolation of *Nocardia* from clinical specimens, including sputum, bronchoalveolar lavage fluid, pleural effusion, or lung tissue specimens. The exclusion criteria were as follows: 1) repeated hospitalization, for which only the first hospitalization was included; and 2) clinical diagnosis without microbiological confirmation; and 3) incomplete key clinical data. This study was approved by the Ethics Committee of Beijing Chaoyang Hospital, Capital Medical University (No. 2025-1097). Due to the retrospective nature of the study, the requirement for written informed consent was waived.

### 2.2 Data collection

Clinical data were retrospectively extracted from the electronic medical record system, including demographic characteristics, clinical symptoms and signs, comorbidities, laboratory test results, radiological manifestations, microbiological findings, treatment, and in-hospital outcomes. Patients were classified into the bronchiectasis group or non-bronchiectasis group according to the presence or absence of bronchiectasis documented in the medical records and chest imaging findings.

### 2.3 Microbiological identification

Clinical specimens, including sputum, bronchoalveolar lavage fluid, pleural effusion, and lung tissue specimens, were submitted to the clinical microbiology laboratory for routine bacterial and fungal culture. Suspected *Nocardia* isolates were preliminarily identified according to colony morphology and microscopic characteristics, including Gram-positive branching filamentous bacteria and weak acid-fast staining. Species-level identification was performed using matrix-assisted laser desorption/ionization time-of-flight mass spectrometry (MALDI-TOF MS; VITEK MS, bioMérieux, France) according to the laboratory workflow and the manufacturer’s instructions. Isolates that could not be reliably assigned to a specific species were reported as unclassified *Nocardia*. Where available, mNGS findings were extracted from the medical records and used as corroborative microbiological evidence.

### 2.4 Statistical analysis

Statistical analysis was performed using SPSS version 22.0 (IBM Corp., Armonk, NY, USA). Continuous variables were expressed as mean ± standard deviation (SD) and compared using Student’s *t*-test. Categorical variables were expressed as numbers and percentages and compared using the chi-square test or Fisher’s exact test, as appropriate. A multivariable binary logistic regression analysis was performed to identify clinical factors independently associated with bronchiectasis among patients with PN. Sex, age, smoking history, fever, cough, expectoration, hemoptysis, respiratory failure, hypertension, type 2 diabetes, chronic bronchitis, bronchial asthma, white blood cell count, and neutrophil count were entered as candidate variables. A backward stepwise method was used for variable selection. A two-sided *P* value <0.05 was considered statistically significant.

## 3 Results

### 3.1 Baseline characteristics

A total of 167 patients with PN-related diagnoses were screened during the study period. Of these, 13 patients were excluded, including 7 repeated hospitalizations, 5 patients diagnosed clinically without microbiological confirmation, and 1 patient with incomplete key clinical data. Finally, 154 patients with PN were included in the analysis.

Among the included patients, 76 were male and 78 were female, with a mean age of 59.74 ± 13.89 years (**Table 1**). The mean length of hospital stay was 14.53 ± 10.06 days. Bronchiectasis was present in 86 patients (55.8%). The most common symptoms were cough (140/154, 90.9%), expectoration (134/154, 87.0%), and fever (84/154, 54.5%), followed by hemoptysis, dyspnea, chest tightness, and chest pain. On physical examination, 100 patients (64.9%) had no positive pulmonary signs, while moist rales were observed in 39 patients (25.3%). Respiratory failure occurred in 27 patients (17.5%), including 21 cases of type I respiratory failure and 6 cases of type II respiratory failure. The most common comorbidities were hypertension (39/154, 25.3%), type 2 diabetes mellitus (23/154, 14.9%), malignancy (21/154, 13.6%), and coronary heart disease (20/154, 13.0%). During hospitalization, 146 patients (94.8%) improved, 5 patients (3.2%) died, and 3 patients (1.9%) were discharged against medical advice.

**Table 1:** Baseline characteristics.

Characteristic	n (%)
Sex (female)	78 (50.6%)
Age (years, mean ± SD)	59.74±13.89
Hospitalization duration (days, mean ± SD)	14.53±10.06
Smoking history	40 (26%)
Symptoms	
Fever	84 (54.5%)
Cough	140 (90.9%)
Expectoration	134 (87%)
Dyspnea	38 (24.7%)
Chest tightness	34 (22.1%)
Chest pain	20 (13.0%)
Hemoptysis	44 (28.6%)
Physical examination	
No positive signs	100 (64.9%)
Wheezing rales	5 (3.2%)
Moist rales	39 (25.3%)
Wheezing and moist rales	10 (6.5%)
Respiratory failure	
Type I	21 (13.6%)
Type II	6 (3.9%)
Comorbidities	
Hypertension	39 (25.3%)
Type 2 diabetes	23 (14.9%)
Coronary heart disease	20 (13.0%)
Malignant tumor	21 (13.6%)
Bronchiectasis	86 (55.8%)
Chronic bronchitis	18 (11.7%)
Bronchial asthma	10 (6.5%)
In-hospital outcomes	
Improvement	146 (94.8%)
Death	5 (3.2%)
Discharge against medical advice	3 (1.9%)

SD, standard deviation.

Among the 154 patients, 53 (34.4%) had microbiological evidence from more than one specimen type or detection method, including 14 with positive cultures from multiple specimen types and 39 with culture findings corroborated by metagenomic next-generation sequencing. A total of 162 *Nocardia* isolates were identified from these patients, including six patients infected with two *Nocardia* species and one patient infected with three species. Among these isolates, the most common species were *N. cyriacigeorgica*, *N. farcinica*, and *N. abscessus*, whereas 29 were not identified to the species level (**Figure 1**). Bacterial co-infection was identified in 44 patients (28.6%), and fungal co-infection was identified in 36 patients (23.4%). Among patients with fungal co-infection, *Candida* species were the most commonly detected fungi.

**Figure 1:**
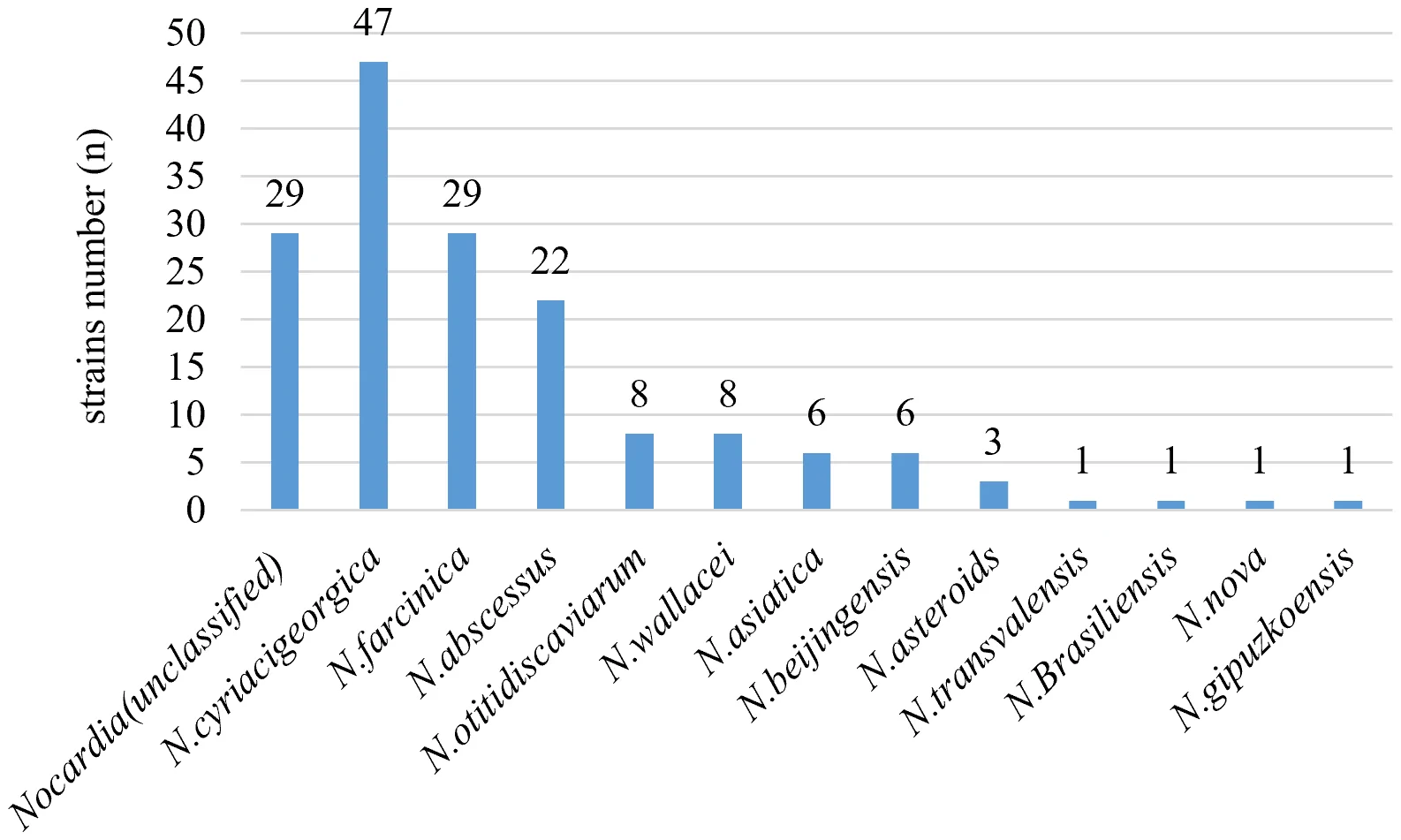
Distribution of *Nocardia* species isolated from patients with pulmonary nocardiosis (PN).

### 3.2 Comparison between patients with and without bronchiectasis

No significant differences were observed between the two groups in age, sex distribution, or smoking history (all *P* > 0.05) (**Table 2**). Most symptoms, including fever, cough, expectoration, dyspnea, chest tightness, and chest pain, did not differ significantly between the two groups (all *P* > 0.05). However, hemoptysis was significantly more frequent in the bronchiectasis group than in the non-bronchiectasis group (40.7% vs. 13.2%, *P*<0.001). In contrast, respiratory failure was significantly more common in the non-bronchiectasis group (26.5% vs. 10.5%, *P*=0.022). Among comorbidities, type 2 diabetes (23.5% vs. 8.1%, *P*=0.008) and bronchial asthma (11.8% vs. 2.3%, *P*=0.018) were significantly more frequent in patients without bronchiectasis. The length of hospital stay was significantly shorter in the bronchiectasis group than in the non-bronchiectasis group (12.62 ± 9.01 vs. 16.96 ± 10.84 days, *P*=0.007). No significant differences were found in bacterial or fungal co-infection and in-hospital death between the two groups (all *P* > 0.05). In the multivariable logistic regression analysis, hemoptysis was positively associated with bronchiectasis [adjusted odds ratio (OR), 5.407; 95% confidence interval (CI), 2.185–13.382; P < 0.01], whereas type 2 diabetes (adjusted OR, 0.320; 95% CI, 0.115–0.888; P=0.029) and bronchial asthma (adjusted OR, 0.166; 95% CI, 0.030–0.928; P=0.041) were negatively associated with bronchiectasis. Smoking history was retained in the final model but did not reach statistical significance (adjusted OR, 0.463; 95% CI, 0.208–1.031; P=0.059) (**Supplementary Table 1**).

**Table 2:** Comparison of clinical characteristics between patients with and without bronchiectasis.

Characteristic	Bronchiectasis group (n=86)	Non-bronchiectasis group (n=68)	*P* value
Age (years, mean ± SD)	58.64±14.23	61.13±13.41	0.270
Sex (female)	38 (44.2%)	38 (55.9%)	0.149
Smoking history	18 (20.9%)	22 (32.4%)	0.108
Symptoms			
Fever	41 (47.7%)	43 (63.2%)	0.054
Cough	78 (90.7%)	62 (91.2%)	0.918
Expectoration	77 (89.5%)	57 (83.8%)	0.295
Dyspnea	17 (19.8%)	21 (30.9%)	0.112
Chest tightness	16 (18.6%)	18 (26.5%)	0.243
Chest pain	11 (12.8%)	9 (13.2%)	0.935
Hemoptysis	35 (40.7%)	9 (13.2%)	<0.001*
Comorbidities			
Respiratory failure	9 (10.5%)	18 (26.5%)	0.022*
Hypertension	17 (19.8%)	22 (32.4%)	0.075
Type 2 diabetes	7 (8.1%)	16 (23.5%)	0.008*
Coronary heart disease	8 (9.3%)	12 (17.6%)	0.126
Bronchial asthma	2 (2.3%)	8 (11.8%)	0.018*
Co-infection			
Bacterial infection	23 (26.7%)	21 (30.9%)	0.572
Fungal infection	16 (18.6%)	20 (29.4%)	0.116
Hospitalization duration (days, mean ± SD)	12.62±9.01	16.96±10.84	0.007*
In-hospital outcome			
In-hospital death	2 (2.3%)	3 (4.4%)	0.655

*P<0.05. SD, standard deviation.

Compared with patients with bronchiectasis, those without bronchiectasis were more likely to present with mass-like shadows (8.8% vs. 0%, *P*=0.005), pleural effusion (32.4% vs. 14.0%, *P*=0.006), pericardial effusion (10.3% vs. 2.3%, *P*=0.036), and hilar lymph node enlargement (16.2% vs. 4.7%, *P*=0.017). In contrast, tree-in-bud signs were more frequent in the bronchiectasis group than in the non-bronchiectasis group (11.6% vs. 2.9%, *P*=0.046). Other imaging findings showed no significant differences between the two groups (all *P* > 0.05) (**Table 3**).

**Table 3:** Comparison of radiological findings between patients with and without bronchiectasis. .

Radiological findings	Bronchiectasis group (n=86)	Non-bronchiectasis group (n=68)	*P* value
Patchy shadow	13 (15.1%)	18 (26.5%)	0.081
Mass shadow	0 (0)	6 (8.8%)	0.005*
Cavitas	9 (10.5%)	8 (11.8%)	0.789
Nodular shadow	24 (27.9%)	25 (36.8%)	0.241
Tree-in-bud	10 (11.6%)	2 (2.9%)	0.046*
Ground-glass opacity	15 (17.4%)	9 (13.2%)	0.475
Cord shadow	27 (31.4%)	19 (27.9%)	0.642
Pleural lesion	50 (58.1%)	38 (55.9%)	0.779
Pleural effusion	12 (14.0%)	22 (32.4%)	0.006*
Pericardial effusion	2 (2.3%)	7 (10.3%)	0.036*
Hilar lymph nodes enlarged	4 (4.7%)	11 (16.2%)	0.017*
Mediastinal lymph nodes enlarged	30 (34.9%)	33 (48.5%)	0.087

*P<0.05.

The four most common *Nocardia* species were the same in both groups, and no significant differences were observed in their proportions (all *P* > 0.05). Among less common species, *N. wallacei* was more frequent in the bronchiectasis group, whereas *N. beijingensis* was more frequently observed in the non-bronchiectasis group, although neither difference reached statistical significance (all *P* > 0.05) (**Figure 2**).

**Figure 2:**
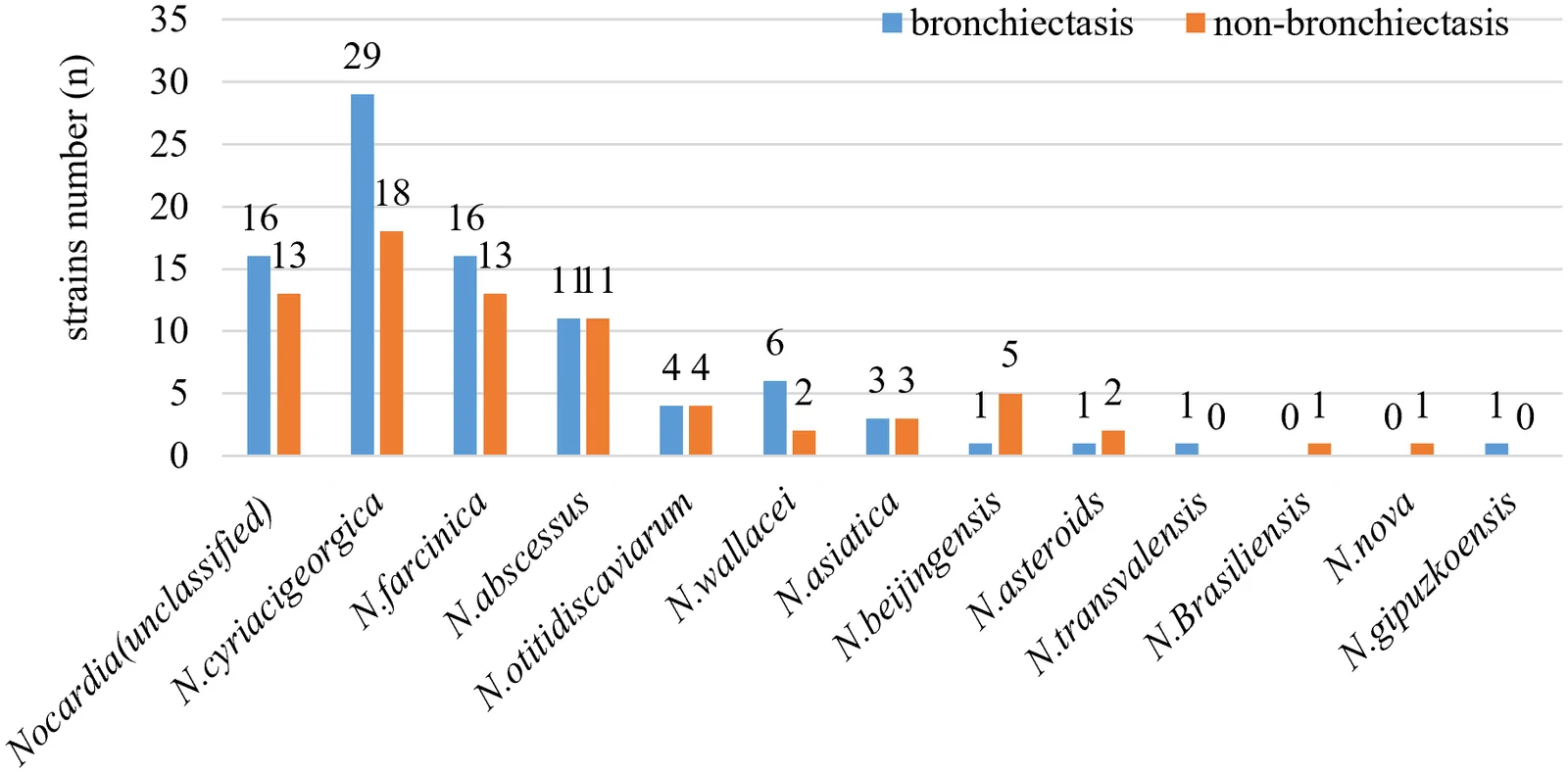
Comparison of *Nocardia* species distribution between PN patients with and without bronchiectasis.

## 4 Discussion

In this retrospective comparative study, we observed several clinical and radiological differences between patients with and without bronchiectasis. Patients with bronchiectasis presented more frequently with hemoptysis, whereas those without bronchiectasis showed higher frequencies of type 2 diabetes, bronchial asthma, respiratory failure, and longer hospital stays. Radiologically, tree-in-bud signs were more common in patients with bronchiectasis, while mass-like shadows, pleural or pericardial effusion, and hilar lymph node enlargement were more frequently observed in patients without bronchiectasis. The overall distribution of major *Nocardia* species was broadly similar between the two groups.

The clinical background and microbiological profile of PN may vary across regions and study populations. In the present study, 55.8% of patients had bronchiectasis, a proportion higher than that reported in several previous cohorts (Woodworth et al., 2017; Zhong et al., 2022; Chen et al., 2024). This finding should be interpreted in the context of the study setting. Our institution is a tertiary respiratory referral center, and the cohort consisted of hospitalized patients from the Department of Respiratory and Critical Care Medicine. Patients with chronic structural lung disease, recurrent respiratory infections, or complex airway conditions may therefore have been preferentially referred or admitted and consequently overrepresented in the study population. Differences in microbiological testing practices may also have contributed to the observed proportion. Accordingly, the prevalence of bronchiectasis in this cohort should not be considered representative of the broader population of patients with PN. In addition, the species distribution in our cohort was generally consistent with recent Chinese epidemiological data, but also showed some differences. Song et al. reported that *N. farcinica*, *N. cyriacigeorgica*, and *N. brasiliensis* were the predominant species in China (Song et al., 2026), whereas the most common identified species in our cohort were *N. cyriacigeorgica*, *N. farcinica*, and *N. abscessus*. This pattern is also broadly consistent with several other previous reports (Valdezate et al., 2017; Wang et al., 2023), although the relative abundance of individual species varied across studies. Notably, *N. beijingensis* was observed more frequently in the non-bronchiectasis group in our cohort; however, this finding should be interpreted with caution because of the small number of isolates. Further studies are needed to determine whether *N. beijingensis* has distinct host preferences or clinical associations. Overall, these findings suggest that both host background and local species distribution should be considered when interpreting the clinical features of PN. Nevertheless, it should be noted that 29 isolates could not be identified to the species level, which may have affected the estimated species distribution, particularly for less common species. The observed differences involving *N. beijingensis* and *N. wallacei* should therefore be interpreted with appropriate caution and confirmed in larger studies with more complete species-level identification.

Notable radiological differences were also observed between the two groups. These differences may reflect different patterns of pulmonary involvement: in patients with bronchiectasis, preexisting airway dilatation, chronic airway inflammation, impaired mucociliary clearance, and fragile bronchial vessels may predispose to airway-centered infection, which could explain the higher frequencies of hemoptysis and tree-in-bud signs. In contrast, patients without bronchiectasis may be less likely to show typical chronic airway changes and may instead present with more parenchymal or pleural inflammatory involvement, such as mass-like opacities, pleural or pericardial effusion, and hilar lymph node enlargement. However, because pathological data were not available, these explanations remain speculative and should be interpreted cautiously.

A recent study by Han et al. also reported differences between *Nocardia*-infected patients with and without bronchiectasis, with a proportion of bronchiectasis comparable to that in the present study (Han et al., 2024). In Han et al (Han et al., 2024), patients without bronchiectasis had a higher treatment failure rate, whereas in our cohort they more frequently developed respiratory failure and had longer hospital stays. These findings indicate that the clinical presentation of PN may differ according to the underlying pulmonary background, although direct comparisons should be made cautiously because of differences in study populations, outcome definitions, and follow-up periods. Additionally, this pattern may be partly explained by earlier medical attention in patients with bronchiectasis due to symptoms such as hemoptysis, whereas patients without bronchiectasis may have a higher burden of systemic risk factors, such as type 2 diabetes and possible asthma-related corticosteroid exposure, and may be diagnosed later because of less specific clinical and radiological manifestations. Therefore, PN in patients without bronchiectasis should be interpreted cautiously, and greater diagnostic attention may still be warranted in this subgroup. Nevertheless, some differences were also observed between the two studies. Han et al. reported significant differences in sex distribution and smoking history between the two groups, whereas these variables were not significantly different in our cohort (Han et al., 2024). In addition, they found that *N. abscessus* was more frequently detected in patients with bronchiectasis and *N. farcinica* in those without bronchiectasis, while the major species distribution in our study was broadly similar between groups. These differences suggest that subgroup-specific patterns of PN may vary across regions and study populations, and should therefore be interpreted cautiously.

This study has several limitations. Firstly, it was a single-center retrospective study with a relatively limited sample size, although this limitation is partly related to the rarity of PN. Therefore, selection bias cannot be excluded, and the generalizability of our findings to other regions or institutions requires further validation. In addition, although multivariable logistic regression was performed for clinical variables, the relatively small number of patients with some characteristics may have affected the stability of the model. Residual confounding from unmeasured factors, such as immunosuppression and other underlying conditions, cannot be excluded. Secondly, due to the retrospective design, some clinically relevant variables were incomplete, such as quantitative inflammatory markers, including procalcitonin and C-reactive protein, detailed diagnosis and treatment information, and prior imaging history. In addition, 101 patients had only a single positive microbiological result; therefore, colonization could not be completely excluded in some cases, particularly among patients with bronchiectasis. Antimicrobial susceptibility testing was also not performed systematically during the study period; therefore, the susceptibility data were unavailable for analysis. Since pre-infection imaging data were not available for all patients, we could not confirm the temporal sequence between bronchiectasis and *Nocardia* infection. Therefore, we could not evaluate species-specific resistance patterns or their potential implications for antimicrobial selection. Thirdly, only in-hospital outcomes were available in this retrospective study, including improvement, death, and discharge against medical advice. Long-term follow-up data were not collected, including recurrence, anti-*Nocardia* treatment duration, long-term pulmonary function changes, and the progression of bronchiectasis after *Nocardia* infection. Therefore, we could not evaluate the long-term prognosis or the potential impact of *Nocardia* infection on subsequent airway structural changes. This limitation should be addressed in future prospective studies with standardized follow-up. Finally, this study did not evaluate any specific diagnostic or therapeutic intervention. Detailed anti-*Nocardia* treatment regimens and duration were not consistently documented, particularly after hospital discharge, and were therefore unavailable for reliable analysis. Consequently, the present findings should not be interpreted as evidence regarding treatment effectiveness or long-term prognosis; instead, they should be considered primarily as evidence supporting improved diagnostic awareness. Future multi-center prospective studies with standardized microbiological, clinical, and follow-up data collection are needed to validate these subgroup-specific features and to develop more effective tools for early diagnosis and differential diagnosis of PN in patients with different underlying pulmonary backgrounds.

## 5 Conclusions

In this cohort, PN showed several clinical and radiological differences between patients with and without bronchiectasis. Hemoptysis and tree-in-bud signs were more frequently observed in patients with bronchiectasis, whereas type 2 diabetes, bronchial asthma, respiratory failure, and longer hospital stay were more frequently observed in patients without bronchiectasis. Further multi-center prospective studies are needed to validate these findings and to support improved early diagnosis and differential diagnosis of PN.

## Data Availability

The raw data supporting the conclusions of this article will be made available by the authors, without undue reservation.
