# Supplementary material for: Clinical characteristics of pulmonary nocardiosis in patients with and without bronchiectasis: a single-center retrospective comparative study
Source: Front Cell Infect Microbiol. 2026 Sep 16;16:1910396. doi: 10.3389/fcimb.2026.1910396 (PMC13623937; doi:10.3389/fcimb.2026.1910396)
Supplement: Supplementary file 1 [file Table1.docx]

**Supplementary Table S1. Multivariable logistic regression analysis of factors associated with bronchiectasis among patients with pulmonary nocardiosis (PN).**

|  | Adjusted OR | 95% CI | *P* value |
| --- | --- | --- | --- |
| Smoking history | 0.463 | 0.208–1.031 | 0.059 |
| Hemoptysis | 5.407 | 2.185–13.382 | <0.01* |
| Type 2 diabetes | 0.320 | 0.115–0.888 | 0.029* |
| Bronchial asthma | 0.166 | 0.030–0.928 | 0.041* |

The presence of bronchiectasis was used as the dependent variable, with the non-bronchiectasis group as the reference category. Candidate variables included sex, age, smoking history, fever, cough, expectoration, hemoptysis, respiratory failure, hypertension, type 2 diabetes, chronic bronchitis, bronchial asthma, white blood cell count, and neutrophil count. Variables retained in the final backward stepwise logistic regression model are presented. OR, odds ratio. CI, confidence interval. **P*<0.05.
